# Application of Ultrasound Combined with Acetic Acid and Peracetic Acid: Microbiological and Physicochemical Quality of Strawberries

**DOI:** 10.3390/molecules26010016

**Published:** 2020-12-22

**Authors:** Priscila Donatti Leão Alvarenga, Christiane Mileib Vasconcelos, Jackline Freitas Brilhante de São José

**Affiliations:** 1 Postgraduate Program in Nutrition and Health, Federal University of Espírito Santo, Marechal Campos Avenue, Vitória, Espírito Santo 28630, Brazil; pridonati@gmail.com; 2 Food Biotechnology Laboratory, Vila Velha University, Comissário José Dantas de Melo Avenue, Vila Velha, Espírito Santo 28630, Brazil; christiane.mileib@uvv.br; 3 Department of Integrated Health Education, Federal University of Espírito Santo, Marechal Campos Avenue, Vitória, Espírito Santo 28630, Brazil

**Keywords:** non-chlorine compounds, disinfection, food quality

## Abstract

This work evaluated the application of organic acids (acetic and peracetic acid) and ultrasound as alternative sanitization methods for improving the microbiological and physicochemical qualities of strawberries. A reduction of up to 2.48 log CFU/g aerobic mesophiles and between 0.89 and 1.45 log CFU/g coliforms at 35 °C was found. For molds and yeasts, significant differences occurred with different treatments and storage time (*p* < 0.05). Ultrasound treatments in combination with peracetic acid and acetic acid allowed a decimal reduction in molds and yeasts (*p* < 0.05). All evaluated treatments promoted a significant reduction in the *Escherichia coli* count (*p* < 0.05). Scanning electron microscopy revealed fragmented *E. coli* cells due to treatment with acetic acid and ultrasound. Storage time significantly affected pH, total titratable acidity, total soluble solids and the ratio of the total titratable acidity to the total soluble solids (*p* < 0.05). Anthocyanin content did not change with treatment or time and generally averaged 13.47 mg anthocyanin/100 g of strawberries on fresh matter. Mass loss was not significantly affected by the applied treatments (*p* > 0.05). The combination of ultrasound and peracetic acid may be an alternative to chlorine-based compounds to ensure microbiological safety without causing significant changes in the physicochemical characteristics of strawberries.

## 1. Introduction

Outbreak records involving the consumption of fresh food contaminated with pathogenic microorganisms highlight the need for improvement in the postharvest sanitization of these products [1,2,3]. Chlorine-based compounds, especially sodium hypochlorite, are commonly used during the sanitization of vegetables to control such outbreaks [4,5,6]. In Brazil, as occurs in other countries, chlorine compounds are the most used sanitizers for fruits and vegetables because they are cheap, easy to prepare and have an antimicrobial efficiency [4,5]. However, there are questions about the efficiency of chlorine-based solutions on biofilms as well as the chemical reactions of free chlorine with organic matter in wash water, which can produce carcinogenic toxic products such as trihalomethanes and haloacetic acids [4,5,6,7]. For this reason, chlorinated compounds are banned in some European countries, such as Germany, Switzerland, the Netherlands, Denmark and Belgium [8,9]. Thus, it is necessary to study alternative strategies to chlorinated compound application that improve the microbiological safety of food without generating toxic byproducts [10,11,12]. The application of peracetic acid, acetic acid, citric acid and lactic acid may be an alternative to a sanitization step [13,14,15,16]. These compounds, which are already widely used in the food industry as preservatives, flavorings, acidulants, antioxidants and pH regulators, are also efficient at inactivating pathogenic microorganisms as sanitizing agents. Peracetic acid has been considered as an alternative use of chlorine in the washing step because of its effectivity against foodborne pathogens and due to it being environmentally friendly [4,5].

Another alternative that has been studied for application in sanitization stage is ultrasound [9,17,18,19,20,21,22,23,24]. This technology is based on high-amplitude waves with frequencies generally above the range of 20 to 50 kHz. The propagation of mechanical vibration causes physical and chemical effects that damage the cell walls of some microorganisms [4,9]. In addition, ultrasound technology has a sanitizing effect that preserves thermosensitive substances and is considered nonthermal [23]. Studies have reported that applications of ultrasound combined with other procedures and/or antimicrobials may increase the inactivation of microorganisms [19,24]. Ultrasound is classed as nonthermal, and it is capable of extending shelf life, decreasing microbial counts and preserving nutritional and sensorial qualities [12], but in some conditions can affect fruit characteristics [6,17].

According to Bastos et al. [6], ultrasound can be an excellent candidate for using on fruits such as strawberries. Strawberry (*Fragaria ananassa* Duch.) is considered a healthy product and is one of the most eaten fruits due nutritional and bioactive properties [6]. However, it is a highly perishable fruit, because of its high contents of water and carbohydrates; as a result, it is extremely probable to have undergone microbiological contaminations from the environment [6,25,26,27]. Besides that, it is susceptible to physical injury and microbial contamination during harvest and transportation [25,26,27]. The epiphytic microbiota of strawberries is varied, and fruits can be contaminated with pathogenic microorganisms from the growth to distribution stage [27]. It is important to mention that an *Escherichia coli* O157:H7 outbreak involved strawberries in the United States in 2011 and caused two deaths [27].

Thus, the aim of this work was to evaluate the application of ultrasound, acetic acid and peracetic acid as alternative treatments for the sanitization of strawberries. The present study investigated the natural microbiota and physicochemical quality of strawberries when subjected to different sanitizing procedures. The proposed treatments were also evaluated when *Escherichia coli* was intentionally inoculated in this fruit. We aimed to verify three hypotheses in this study: (i) acetic and peracetic acid would show similar or better effectiveness in the disinfection than the chlorine-based sanitizer; (ii) ultrasound in combination with acetic acid and peracetic acid would improve the chemical sanitizer efficiency; (iii) the treatments would contribute to the preservation of physicochemical properties in storage.

## 2. Materials and Methods

### 2.1. Sample Preparation and Sanitization Treatments

Strawberry samples were obtained from a local market in Cariacica, Espírito Santo, Brazil. After acquisition, the samples were immediately transported in isothermal boxes to a laboratory to begin the analyses. The fruits that presented the same maturation stage were selected and those with deformities or damage were discarded. First, the peduncle and calyx were removed and then all strawberry samples were washed in running water to remove soil adhered to the surface and then were drained at room temperature for 10 min. After, strawberries underwent the sanitization step with proposal treatments. The treatments were as follows: sodium hypochlorite sanitizer solution prepared with Hidrosteril^®^ (Indaiatuba, SP, Brazil) at 1 mL/L, ultrasound 40 kHz/110 W, Branson^®^, Model CPX3800 H (Danbury, United States) at 15 °C (equipment has temperature control, tank capacity 5.7 L and tank size 290 × 150 × 150 mm), 10 mL/L acetic acid solution (Vetec^®^, São Paulo, SP, Brazil), 2 mL/L peracetic acid solution prepared with Nippo-Lat 2000 AP (Nippon Chemical^®^, Indaiatuba, SP) Brazil), 10 mL/L acetic acid solution combined with 40 kHz ultrasound and 2 mL/L peracetic acid solution combined with 40 kHz ultrasound. The temperature was monitored every 1 min to check for possible variations. Samples that were treated only by ultrasound were immersed in water. Untreated samples were used as control.

The sanitization step consisted of immersing the strawberries in each sanitizing solution at a 1:10 ratio (100 g of strawberries per L of solution) for 5 min at 15 ± 1 °C. After the treatments, the strawberries were drained for 10 min and then were refrigerated in disinfected polyethylene terephthalate packaging at 6 ± 1 °C. Strawberry samples were analyzed immediately after sanitization (day 1), and at 3, 6 and 9 days of storage.

In addition to the analysis of the effects on the physicochemical quality and natural microbiota contaminants on strawberries, sanitization treatments were evaluated on fruits intentionally inoculated with *Escherichia coli.*

### 2.2. Physicochemical Analysis

These analyses aimed to identify changes in the quality of fruit after the sanitization treatments compared to the quality of untreated samples (control). The samples used in this series of tests were not inoculated with microorganisms. The total titratable acidity, pH, total soluble solids, and vitamin C content were determined according to the Association of Official Analytical Chemists [28]. The determination of vitamin C was carried out by titration by Tillmans’ method [28], which is based on the reduction in the sodium salt of the dye 2,6-dichlorophenol indophenol by an acidic solution of vitamin C, and resulted in the mg of ascorbic acid contained in 100 g of strawberries. Anthocyanin was determined following the methodology proposed by Francis [29]. An extract was prepared using 1 g of sample and 60 mL of methanol/water (70:30 *v*/*v*), and the pH was adjusted to 2. This extract was stored in amber glass for 24 h at 3 °C. The extract was then vacuum-filtered and diluted (1:10 *v*/*v*) in 1.5 mol/L methanol-HCl (85:15%). A spectrophotometer was then read at a wavelength of 535 nm. The anthocyanin content is expressed as the amount of cyanidin-3-glycoside. The mass loss for each storage time was calculated based on the initial weight on day 1. Each package containing strawberries was weighed on a AS-5500 C precision scale balance (Marte^®^, São Paulo, SP, Brazil). The results were expressed as a percentage of the total mass loss (%).

### 2.3. Evaluation of Natural Microbiota

The procedures used in this step were performed according to the methodology described in the Compendium of Methods for the Microbiological Examination of Foods [30]. Analyses were conducted for aerobic mesophile bacteria, molds and yeasts and coliforms at 35 °C and for *Escherichia coli.* Strawberry samples (25 g) were homogenized with 225 mL of 0.1% peptone water in a stomacher (YK Tecnologia^®^, Sapiranga, RS, Brazil) for 2 min at normal speed. Appropriate serial dilutions were prepared for inoculation in standard plate count agar (PCA) (Acumedia^®^, Indaiatuba, SP, Brazil), which was incubated for 48 h at 35 ± 1 °C to determine aerobic mesophile abundance. For molds and yeasts, aliquots were inoculated on potato dextrose agar (PDA) (Acumedia^®^, Indaiatuba, SP, Brazil) at pH 3.5 and incubated at 25 ± 2 °C for 5 to 7 days. For coliforms, incubations at 35 °C were carried out with Petrifilm dishes *Escherichia coli* (EC) (3M^®^, Sumaré, SP, Brazil) following the manufacturer’s instructions. After incubation of the plates at 35 °C for 24 and 48 h, blue and red colonies with gas were considered colonies of total coliforms, and blue colonies with gas were considered *E. coli*. The aliquots were plated in duplicate, and the results were expressed in colony forming units per gram (CFU/g).

### 2.4. Challenge Test with Escherichia coli ATCC 11229

Samples of strawberries were obtained as described in Section 2.1, selected and washed with sterile distilled water. *E. coli* ATCC 11229 was obtained from the culture stock of the Microbiology Laboratory of the Federal University of Espírito Santo. The culture was maintained in 1 mL microtubes (Eppendorf^®^, São Paulo, Brazil) containing Brain Heart Infusion (BHI) agar (Difco^®^, Sparks, MD, USA) at −80 °C and activated by two consecutive BHI replications and incubation at 37 °C for 24 h until reaching populations of 10^8^ and 10^9^ CFU/mL.

To evaluate the effect of the treatments, inoculation of 100 g of strawberries was performed in previously sterilized plastic bags, and 200 mL of 0.1% peptone water was added to the inoculum (10 mL) to give a solution with approximately 10^6^ CFU/mL. The plastic bag containing the inoculum and the sample was shaken gently for 5 min. The strawberries were kept in static contact with the cell suspension for 60 min at 24 ± 1 °C. Then, the cell suspension was drained, and the intentionally inoculated strawberries were subjected to sanitization treatments. The sanitization procedures were conducted as described in Section 2.1. Samples to be treated with ultrasound were placed in previously sterilized plastic bags that were then placed inside the equipment. The purpose of this procedure was to prevent contamination of the equipment with the microorganism. After each treatment, 25 g of strawberries was transferred to sterile plastic bags with 225 mL of 0.1% peptone water and then homogenized for two minutes. Thereafter, appropriate dilutions were prepared and plated on MacConkey agar (Acumedia^®^, Indaiatuba, SP, Brazil). After plating and incubation for 48 h at 35 °C, colonies were counted.

### 2.5. Scanning Electron Microscopy (SEM) of Strawberries Intentionally Inoculated with Escherichia coli ATCC 11229

Samples were prepared for SEM according to the method described in Section 2.1. Briefly, 1.0 cm × 1.0 cm pieces were prepared using a previously sterilized scalpel. SEM of the surface of strawberries inoculated with *E. coli* was performed according to the method described by São José and Vanetti [4]. The samples were dried in a critical point dryer model autosamdri-815 (Tousimis^®^, Maryland, USA). After this, the samples were placed in a Desk V sputter coater (Denton Vacuum^®^, Cherry Hill, NJ, USA) for deposition of a thin layer of gold and then analyzed in scanning electron microscopic model JSM-6610 SEM LV (JEOL^®^, Tokyo, Japan).

### 2.6. Statistical Analyses

To compare the effects of sanitization treatments on the reduction in the natural microbiota and physicochemical values, analyses were performed at days 1, 3, 6 and 9 of storage at 6 °C. The results were submitted to a two-way analysis of variance (ANOVA) to verify the significance of the factors in the sanitization treatment and storage time and the interaction between them. All experiments were carried out in three repetitions. To evaluate the reduction in *E. coli*, the analyses were performed only immediately after sanitization, and one-way ANOVA was used. Data were submitted to ANOVA, Pearson’s correlation test and Duncan’s test at the 5% probability level and the student version of InfoStat^®^ software (Córdoba, Argentina) were used.

## 3. Results and Discussion

### 3.1. Effect of The Sanitizing Treatments on the Physicochemical Characteristics of Strawberries

Treatments had a similar behavior throughout storage time. The storage time significantly affected the pH, total titratable acidity, total soluble solids’ values and ratio of the total titratable acidity of the total soluble solids (*p* < 0.05) (Table 1).

**Figure 1:**
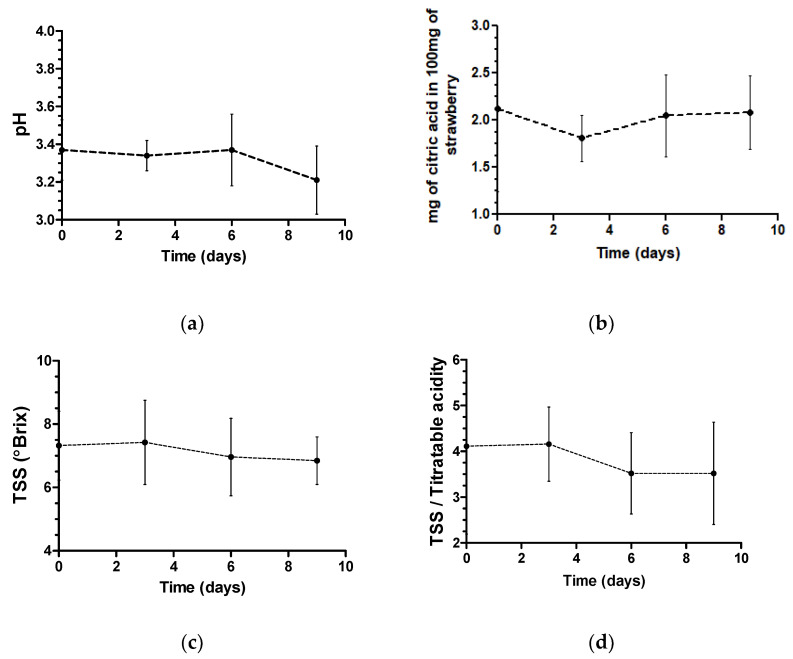
Mean and standard deviation of pH (**a**), total titratable acidity (**b**), total soluble solids (**c**) and total soluble solids/total titratable acidity (**d**) were evaluated in strawberries of all treatments and control during 9 d of storage.

However, since it was not possible to fit these parameters into a linear or quadratic equation as a function of time, a graph was designed to understand the parameters’ behaviors (Figure 1).

The overall mean pH of all treatments was 3.37 on the first day and 3.21 on the ninth day. The average pH in all the treatments and the control initially decreased and can be associated with the application of organic acids since they dissociate to form hydronium ions, reducing the pH of the solution [31]. Beginning on the sixth day, there was a decline in pH in all treatments and in untreated strawberries, which, according to Rahman, Moniruzzaman, Ahmad, Sarke and Alam [32], may be associated with strawberry senescence. As in this study, Rosário et al. [24] also did not observe a significant change in pH under different treatments (acetic acid and peracetic acid) or with increasing storage time, reporting a mean pH of 3.47. However, in the São José and Vanetti [33] study, they observed lower pH values in strawberries treated with peracetic acid for 10 min than in untreated strawberries and this can be associated with sanitization duration.

The ratio of total soluble solids to total titratable acidity decreased during storage, showing that the treatments may have contributed to the acceleration of strawberry quality loss. The ratio between total soluble solids and acidity, being more representative than the isolated measures of sugar content or acidity, is an important metric of fruit quality and is used for flavor evaluation. This relationship demonstrates the balance between these two components [34] and may be associated with the sensorial characteristics of foods, revealing the degree of maturation of the raw material.

**Figure 2:**
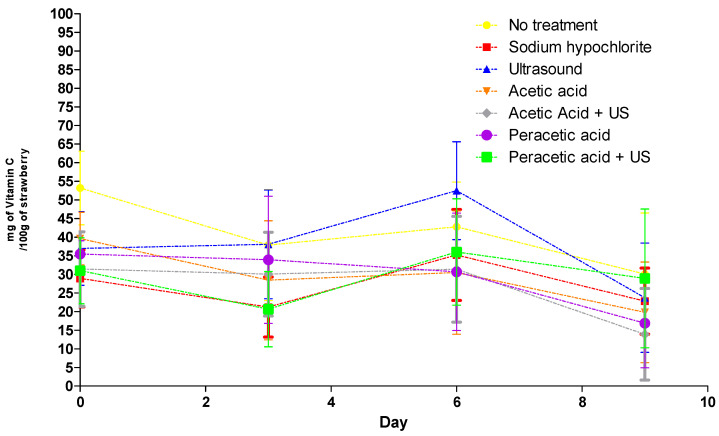
Mean variation of vitamin C analyzed in strawberries from all treatments and control during 9 d of storage.

With regard to vitamin C levels, a significant interaction between time and treatment was observed and investigated further to elucidate their individual effects (Figure 2).

Linear and quadratic models were tested to explain the behavior of the analyzed variables as a function of time; however, it was not possible to remove significant adjustment and obtain significant regression models. Thus, the variables were plotted to visualize their behavior. After the treatments were applied to the samples on the first day, the vitamin C content decreased until the third day (Figure 2), except in the sample treated with ultrasound. Vitamin C is highly thermolabile; therefore, this compound is extremely sensitive to chemical and enzymatic oxidation and soluble in water, making it very sensitive to certain postharvest treatments [35].

Regarding the application of ultrasound and its impacts on the vitamin C content, it is likely that the hot spots formed during cavitation were not sufficient to cause vitamin C degradation. In ultrasound, retention of bioactive compounds can be attributed to a reduction in dissolved oxygen due to cavitation or to an inactivation of enzymes that lead to reduced vitamin contents [36].

Between the third and sixth days, there was an overall increase in vitamin C content in the treatments. Ascorbic acid is involved in the regulation of several key cellular processes, such as photoprotection, cell cycle, cell expansion and secondary metabolism pathways such as the recycling of liposoluble α-tocopherol and ethylene biosynthesis; therefore, vitamin C may have been synthesized as a defense against oxidative stress, since the accumulation of ascorbic acid in tissues and organs of the plant is altered by physiological phenomena, such as senescence and cell development and expansion, and various biotic and abiotic stimuli [37,38,39]. After the sixth day, the level of ascorbic acid dropped, which may have been due to the death of cells in the fruit tissues, which paralyzed the synthesis of ascorbic acid, together with an acceleration of the oxidation of this compound.

Rosário et al. [5] observed different results and detected that treatments (acetic acid; sodium dodecylbenzenesulfonate; peracetic acid; ultrasound) and the interaction (treatment × storage time) did not significantly affect vitamin C in strawberries. The maintenance of vitamin C in ready-to-eat products is important since consumers want fresh and healthy products.

The anthocyanin content did not change with changes in treatment or time (*p* > 0.05), exhibiting an average of 13.47 mg anthocyanin/100 g sample. Thus, the lack of differences between the treatments indicates that anthocyanins were preserved in samples subjected to these treatments. Different results were observed by Gani et al. [36] when they evaluated the effect of ultrasound during 60 min and this did not result in better color retention during storage. It is important to mention that anthocyanin contributed to food color and nutritional value. The concentration of anthocyanins in fruits is increased during the ripening stage, when the biosynthetic rate is accelerated due to the action of ethylene, which triggers the activation of many enzymes involved in anthocyanin biosynthesis and storage at low temperatures does not inhibit this process [36]. Ultrasound and sanitizers affect the superficial regions of the strawberry; the internal anthocyanin content of strawberries might not be affected and thus is likely preserved during storage.

Weight loss was not significantly affected by the applied treatments (*p* > 0.05). The weight of strawberries without sanitization decreased by 0.62 ± 0.23%, and the weight of treated strawberries decreased by 0.36% to 0.63% between the first and last day of storage. Fruit weight loss is mainly linked to respiration and evaporation of moisture through the skin. The samples were stored at low temperature; therefore, there was not a high rate of evaporation for the moisture in the fruits. When excess water is lost by the vegetal tissue due to transpiration, the loss of mass can increase, which leads to a decrease in nutritional content and modifications to pulp firmness and aroma [40].

The pH was significantly and negatively correlated (*p* < 0.05) with the total titratable acidity and with the ratio of the acidity to the total soluble solids (Table 2). The total soluble solids content positively correlated with the pH and acidity (*p* < 0.05). When strawberry senescence occurs, the acidity decreases; consequently, the pH increases, and sucrose is hydrolyzed to fructose and glucose, which contributes to the increase in total soluble solids [41]. A correlation between the total soluble solids and anthocyanin contents was significant and positive and can be explained by the fact that during senescence of the fruit, anthocyanin production occurred with the production of sugars from sucrose degradation. The ratio of the acidity to the total soluble solids was inversely proportional to the vitamin C content since the soluble solids increase, fruit acidity decrease, and vitamin C increase reflect the acidic composition of the fruit.

### 3.2. Effect of the Sanitization Treatments on Natural Microbiota

After the aerobic mesophiles and coliforms were analyzed at 35 °C, the effects of the applied sanitization treatments were verified to be not significantly different (*p* > 0.05). Time did not significantly affect the coliform counts. In addition, there was no significant relationship between the storage time and treatments for aerobic mesophiles, coliforms, molds and yeasts (Table 3).

Application of the strawberry sanitization treatments reduced the aerobic mesophile count by 1.09 to 2.48 log CFU/g in strawberries (Table 4). São José and Vanetti [33] evaluated the application of ultrasonic sanitization in combination with 40 mg/L peracetic acid for 10 min, and a reduction of 4.1 log CFU/g was observed in the number of aerobic mesophile bacteria on strawberries. A comparison of these results with those of the present study indicated that the increase in the peracetic acid concentration together with the increase in the exposure time of this acid on the strawberry results in an increase in the reduction in aerobic mesophiles. In the same way, the application of ultrasound of increased frequency for more time demonstrated improved results.

**Figure 3:**
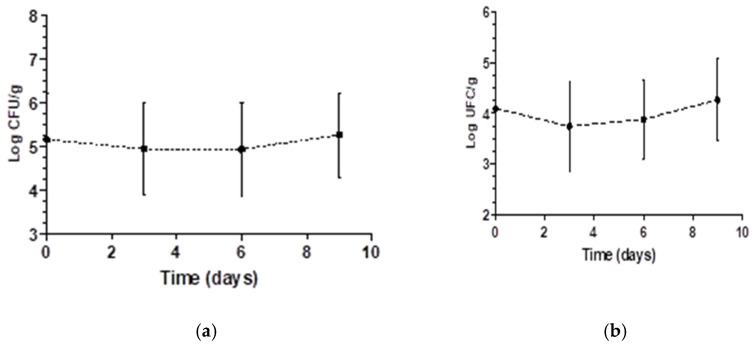
Mean overall variation of the count of aerobic mesophiles (**a**) and molds and yeasts (**b**) in log of the CFU/g evaluated in strawberries of each treatment during 9 days of storage.

The storage time affected the aerobic mesophile count; thus, these two variables were plotted to assess the behavior during storage (Figure 3a).

The average initial aerobic mesophile count was 5.16 log CFU/g, with decay occurring until the third day, potentially due to the reduced pH in the samples treated with organic acids. Some aerobic mesophiles grow at low pH, contributing to the production of acidic substances (lactic acid, acetic acid and others) and the development of unpleasant flavors [42]. Regarding the coliform counts at 35 °C, there were no significant differences among the treatments or over time and no interactions between the investigated variables, and reductions between 0.89 and 1.45 log CFU/g were observed after the sanitization treatments.

For molds and yeasts, a significant difference was observed with treatment and time (Table 4). Thus, these variables were studied separately. To study the effects of the different treatments, a comparison test of means was performed. The application of ultrasonic treatments in combination with peracetic acid and acetic acid resulted in an increased decimal reduction in molds and yeasts (*p* < 0.05). The results of the combined ultrasound and acetic and peracetic acid treatments were significantly different from those of the sodium hypochlorite treatment. These results indicate that the proposed sanitizers are more efficient at controlling this microbial group than the conventional treatment. Nicolau-Lapena et al. [26] observed a different result for yeasts and molds: treatment with chlorine compounds and peracetic acid reduced contamination in washing water, but no differences were observed in strawberries.

Storage time significantly affected mold and yeast counts (*p* < 0.05). However, since it was not possible to fit a linear or quadratic equation for the mold and yeast count data, the mean and standard deviation as a function of storage time were plotted (Figure 3b). The decay of molds and yeasts between days 1 and 3 can be explained by the effects of the treatments. At the end of the storage period, growth occurred, which may have been due to the decrease in pH. Molds and yeasts tolerate lower pH values better than bacteria and are associated with the deterioration of acidified foods and products made from acidic fruits. Spore survival under the treatments may have also enabled the mold and yeast growth [43].

### 3.3. Effect of the Treatments on Escherichia coli ATCC 11229 Adhered on the Strawberry’s Surface

All evaluated treatments promoted a significant reduction in the initial *E. coli* count (*p* < 0.05) (Table 5). The treatments with peracetic acid, acetic acid, sodium hypochlorite, ultrasound and ultrasound combined with acetic acid promoted equivalent reductions (*p* > 0.05). This result indicated that the efficacies of these treatments are equivalent; therefore, treatments with peracetic acid, acetic acid, ultrasound, and ultrasound combined with acetic acid may replace treatment with sodium hypochlorite for the inactivation of *E. coli* cells.

Treatment with ultrasound combined with peracetic acid promoted a greater reduction in the counts of *E. coli* adhered to the surface of strawberries (*p* < 0.05). Treatment with peracetic acid alone and combined with ultrasound showed the greatest decrease in pH in the first days of treatment, which may have caused the increased reduction in *E. coli* [44]. Gurtler, Bailey, Jin and Fan [45] obtained similar results in the evaluation of the effects of 1% acetic acid solution treatment for 2 min on seven strains of *E. coli* O157:H7 on inoculated strawberries. Nicolau-Lapena et al. [26] artificially inoculated *Listeria innocua* in strawberries and observed that all sanitizantion treatments with peracetic acid reduced its number by at least 4 log CFU/g, except for the 20 mg/L treatment for 1 min.

#### Scanning Electron Microscopy

**Figure 4:**
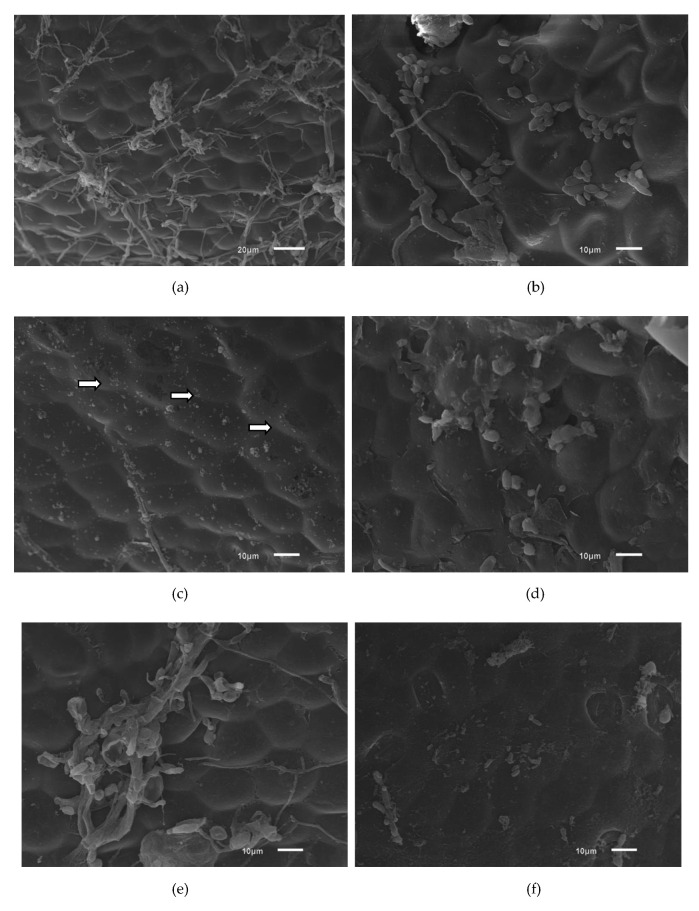

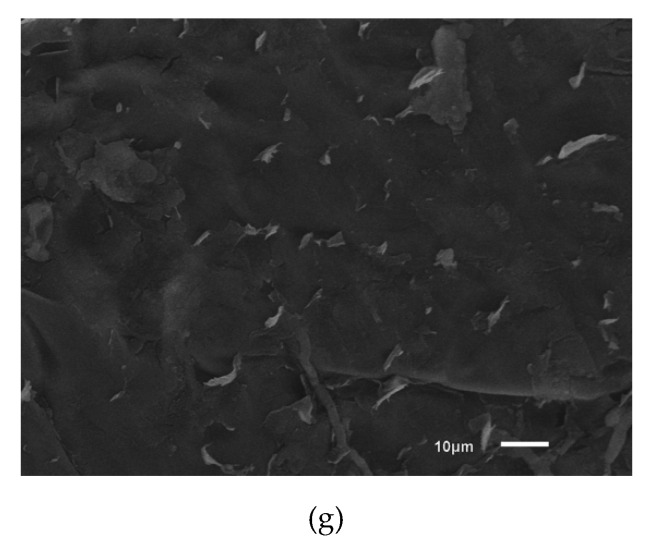
Photomicrographs of strawberry sections intentionally contaminated with ATCC 11229 *E. coli* cells using different sanitation procedures for 5 min and observed by scanning electron microscopy (3000×). (**a**) No treatment; (**b**) Sodium hypochlorite 1 mL/L; (**c**) Ultrasound 40 kHz; (**d**) Acetic acid 10 mL/L; (**e**) Acetic acid 10 mL/L and Ultrasound 40 kHz; (**f**) Peracetic acid 2 mL/L; (**g**) 2 mL/L peracetic acid and 40 kHz ultrasound.

As shown in Figure 4a, the strawberry structure has a roughness that may favor bacterial adhesion. Increased mean roughness of a surface can favor the retention of microorganisms [46]. In Figure 4b, intact *E. coli* cells can be observed. In Figure 4c, the cells are fragmented (white arrows), indicating damage caused by ultrasound treatment via the irregular collapse of cavitation bubbles, which caused erosion of the cell walls, resulting in the inactivation of the microorganisms [23].

Figure 4d shows *E. coli* cells with changes to their morphology that may be related to the treatment with acetic acid since this acid is lipophilic and can cross the cytoplasmic membrane, destabilize the intracellular pH of the microorganism and cause cell disintegration [47]. In Figure 4e, the microbial cells are disrupted and exhibit morphological alterations. Acidification used to control the growth of microorganisms may lead to changes in bacterial cell morphology [48].

Notably, *E. coli* cells were completely fragmented by treatment with acetic acid and ultrasound (Figure 4e). In the treatments with peracetic acid combined with ultrasound (Figure 4 g), a reduced number of microorganisms on the strawberry surface, an altered morphology and fragments of *E. coli* cells were observed. Peracetic acid releases singlet oxygen, which is a low-molecular-weight compound that can cross the bacterial membrane, react with internal cellular components, and cause damage to cellular structures and the release of intracellular components [49].

According to Firouz et al. [50], some corporations expertly work on the commercialization of ultrasound for application in the food industry. It is important to mention that in the industry high volumes of material must be treated, contrasted to laboratory methods. Ultrasonic baths with agitation systems can be applied and this method can replace many of the conventional procedures in food processing [50]. Ultrasound is classified as nonthermal, and it is proficient in extending the shelf life and reducing microbial counts [12]. This method has potential in the food industry and can be useful in fast-tracking practices, decreasing energy supplies, improving productivity, and producing food products with good quality [50].

One limitation of this study can be considered in its evaluation of only one frequency and time of application of the ultrasound alone or combined with sanitizing agents to better understand the effects of treatments on strawberries. It is important to mention that commercial information of ultrasound application is scarce, the radiation area of transducers could be a problem for the food industry and each type of food present different characteristics. Then, application of ultrasound must be a challenge and it is a necessary effort to advance on large-scale equipment [51].

## 4. Conclusions

Ultrasound is a novel technology and in the last years received attention in research related to food processing and preservation. Among the treatments applied, the treatments in which ultrasound was combined with peracetic acid and acetic acid yielded greater efficiency in the inactivation of molds and yeasts and of *E. coli* cells intentionally inoculated on the surface of strawberries. All applied disinfection treatments preserved the physicochemical characteristics of strawberries, indicating that the proposed sanitizers, especially the combined treatments, have the potential to replace the chlorinated compounds commonly used in sanitation. It is important to mention that in addition of inactivate spoilage microorganisms, as well as pathogenic bacteria, is crucial to preserving the physicochemical and nutritional properties of the food.

This research was conducted under controlled laboratory conditions and it is difficult to say that the same results would be observed on an industrial scale. So, further research is required to clarify all aspects.

## Figures and Tables

**Table 1:** Analysis of variance (ANOVA) for pH, total soluble solids, total titratable acidity, total soluble solids/total titratable acidity ratio, vitamin C and anthocyanins in sanitized strawberries in storage for 9 days at 6 ± 1 °C.

Variables	Variation Sources	Degrees of Freedom	F Value	*p* Value
pH	Treatment	6	0.36 n.s.	0.8917
	Time	3	8.23 *	0.0003
	Interaction	18	0.07 n.s.	1.000
Total soluble solids	Treatment	6	2.27 n.s.	0.0967
	Time	3	2.90 *	0.0491
	Interaction	18	1.19 n.s.	0.3248
Total titratable acidity	Treatment	6	0.26 n.s.	0.9459
	Time	3	9.38 *	0.0001
	Interaction	18	0.10 n.s.	1.0000
Total soluble solids/total titratable acidity ratio	Treatment	6	0.38 n.s.	0.8797
	Time	3	8.72 *	0.0002
	Interaction	18	0.43 n.s.	0.9714
Vitamin C	Treatment	6	2.43 n.s.	0.0806
	Time	3	2.33 n.s.	0.0925
	Interaction	18	6.76 *	0.0001
Anthocyanins	Treatment	6	0.42 n.s.	0.8503
	Time	3	1.05 n.s.	0.3897
	Interaction	18	0.53 n.s.	0.8808

* Significant F value (*p* < 0.05) after analysis of variance. n.s.: F value not significant (*p* > 0.05) after analysis of variance.

**Table 2:** Correlation coefficient between the physical–chemical parameters of strawberries submitted to different sanitization procedures.

	pH	Total Titratable Acidity (TTA)	Total Soluble Solids (TSS)	TSS/TTA	Vitamin C	Anthocyanins
pH		−0.8335 *	0.2992 *	−0.7184 *	0.14832	0.3901 *
TTA			−0.2588 *	0.7670 *	−0.1665	−0.4835 *
TTS				−0.1115	−0.1378	0.4153 *
TTS/TTA					−0.2292 *	−0.4226 *
Vitamin C						0.1792
Anthocyanins						

* Significant considered alpha = 0.05.

**Table 3:** Summary of analysis of variance (ANOVA) of the count of aerobic mesophiles, molds and yeasts and coliforms at 35 °C in strawberries during storage of 9 days at 6 ± 1 °C.

Variables	Variation Sources	Degrees of Freedom	F Value	*p* Value
Aerobic mesophiles	Treatment	6	2.24 n.s.	0.6232
	Time	3	0.55 *	0.0288
	Interaction	18	0.11 n.s.	0.7144
Coliforms 35 °C	Treatment	6	1.70 n.s.	0.1573
	Time	3	1.45 n.s.	0.2505
	Interaction	18	0.38 n.s.	0.9817
Molds and yeasts	Treatment	6	7.51 *	0.0089
	Time	3	5.75 *	0.0049
	Interaction	18	0.29 n.s.	0.8952

* Significant *p*-value (*p* ≤ 0.05). n.s.: *p* value not significant (*p* ≥ 0.05).

**Table 4:** Mean aerobic mesophiles, molds and yeasts and coliform at 35 °C counts (log CFU/g) in strawberries not treated and submitted to different sanitization procedures and stored for 9 days at 6 ± 1 °C.

Treatment	Aerobic Mesophiles	Molds and Yeasts	Coliforms at 35 °C
No treatment	6.40 ± 0.50a	5.07 ± 0.52a	4.30 ± 0.75a
Sodium hypochlorite 1 mL/L	5.31 ± 0.74a	4.20 ± 0.60b	3.27 ± 0.92a
Acetic acid 10 mL/L	5.18 ± 0.79a	3.97 ± 0.66b	3.03 ± 0.64a
Peracetic acid 2 mL/L	4.86 ± 0.82a	3.89 ± 0.60b	2.99 ± 0.73a
Ultrasound 40 kHz	5.34 ± 0.66a	4.31 ± 0.49b	3.41 ± 0.69a
Ultrasound 40 kHz + Acetic acid 10 mL/L	4.36 ± 0.84a	3.34 ± 0.35c	2.98 ± 0.72a
Ultrasound 40 kHz + Peracetic acid 2 mL/L	3.92 ± 0.81a	3.08 ± 0.33c	2.85 ± 0.57a

**Table 5:** *Escherichia coli* ATCC 11229 count in log CFU/g adhered to strawberry’s surface (no treatment) and after being submitted to different procedures of sanitization.

Treatment	Log CFU/g	Reduction Log CFU/g
No treatment	5.38 ± 0.56a	–
Sodium hypochlorite 1 mL/L	4.39 ± 0.29b	0.99
Acetic acid 10 mL/L	4.12 ± 0.42b	1.26
Peracetic acid 2 mL/L	4.30 ± 0.49b	1.08
Ultrasound 40 kHz	4.34 ± 0.17b	1.04
Ultrasound 40 kHz + Acetic acid 10 mL/L	3.92 ± 0.58bc	1.46
Ultrasound 40 kHz + Peracetic acid 2 mL/L	3.17 ± 0.41c	2.21

## Sample Availability

Samples of the compounds are not available from the authors.
